# From suspected necrotizing fasciitis to diagnosed drug-induced fever: a diagnostic maze and reflections on anti-infection management in a case of postoperative fever after femoral fracture

**DOI:** 10.3389/fmed.2026.1830157

**Published:** 2026-07-14

**Authors:** Yaoxin Ao, Lili Duan, Jiaomei Shi, Yanjiao Li, Huaxi Sun, Sijie Liu, Can Qu, Bixia Yuan, Xinguo Zhang

**Affiliations:** 1 Department of Orthopedics, Shenzhen Hospital (Futian) of Guangzhou University of Chinese Medicine, Guangzhou University of Chinese Medicine, Shenzhen, China; 2 Department of Pediatrics, The University of Hong Kong-Shenzhen Hospital, Shenzhen, China

**Keywords:** antimicrobial stewardship, case reports, drug fever, femoral fractures, necrotizing fasciitis, piperacillin–tazobactam, vancomycin

## Abstract

Postoperative fever represents a common clinical challenge in orthopedics, and the differential diagnosis of its etiology directly influences anti-infective decision-making and patient outcomes. In the context of anti-infective therapy, “drug fever” induced by antimicrobial agents themselves can easily be mistaken for uncontrolled or recurrent infection, leading to antibiotic overuse. This article reports the case of a 49-year-old male with a comminuted fracture of the right femur. Preoperatively, he presented with post-traumatic absorption fever. Postoperatively, due to intraoperative observation of fascial necrosis and a sharp rise in inflammatory markers, a high clinical suspicion of necrotizing fasciitis prompted the initiation of triple broad-spectrum antibiotic therapy (clindamycin + piperacillin-tazobactam + vancomycin). After initial infection control, a regular afternoon high-grade fever emerged in the second postoperative week, accompanied by a “vancomycin infusion syndrome”-like rash and agranulocytosis. Through systematic exclusion of infectious sources and a rigorous drug withdrawal trial, the final diagnosis was confirmed as “antimicrobial-associated drug fever.” This case comprehensively illustrates the three-stage dynamic evolution of perioperative fever: from “traumatic inflammation” to “bacterial infection” and finally to “drug fever.” It highlights that clinicians should maintain high vigilance during active anti-infective treatment for the sequential phenomenon whereby “the therapeutic agents themselves become a new source of fever after infection control,” in order to avoid inappropriate prolongation of antibiotics due to misjudgment and to reinforce the principles of antimicrobial stewardship.

## Introduction

Postoperative fever following fracture surgery represents a core management challenge routinely encountered by orthopedic surgeons. Its etiological spectrum is broad, encompassing non-infectious systemic inflammatory responses triggered by surgical trauma or hematoma absorption, to life-threatening infectious complications such as surgical site infection, implant-related infection, and necrotizing fasciitis (NF). Among these, NF, due to its rapid progression and high mortality rate, is often regarded as the “primary threat” requiring urgent exclusion during differential diagnosis. This vigilance frequently drives clinicians to initiate empiric therapy with broad-spectrum antibiotics. However, a frequently underestimated clinical reality is that the antimicrobial agents used to treat infections can themselves become a new source of fever, namely drug fever. Drug fever is typically induced by a T-cell-mediated delayed-type (type IV) hypersensitivity reaction, and its onset is closely related to the duration of drug exposure, potentially accompanied by manifestations such as rash and eosinophilia (1). When drug fever superimposes on an incompletely resolved infectious fever course, it is prone to lead to a misdiagnosis of “uncontrolled infection” or “infection recurrence.” This can trap clinicians in a cycle of blindly escalating or prolonging antibiotic therapy, which not only increases the patient’s risk of adverse effects (e.g., bone marrow suppression, hepatorenal injury) and secondary infections with drug-resistant organisms but also deviates from the core principles of “precise management and timely de-escalation” in contemporary anti-infective therapy.

Therefore, enhancing the recognition of perioperative drug fever is crucial for optimizing outcomes in orthopedic patients and promoting rational antibiotic use. By detailing a case of femoral fracture that progressed from high suspicion of necrotizing fasciitis to a final diagnosis of drug fever associated with piperacillin-tazobactam and vancomycin, this article aims to illuminate the diagnostic pitfalls in this clinical scenario. It further encourages profound reflection on anti-infective management strategies, intending to provide valuable insights for clinical practice.

## Case presentation

### Admission and preoperative period (July 31, 2025 to August 10, 2025)

**Figure 1:**
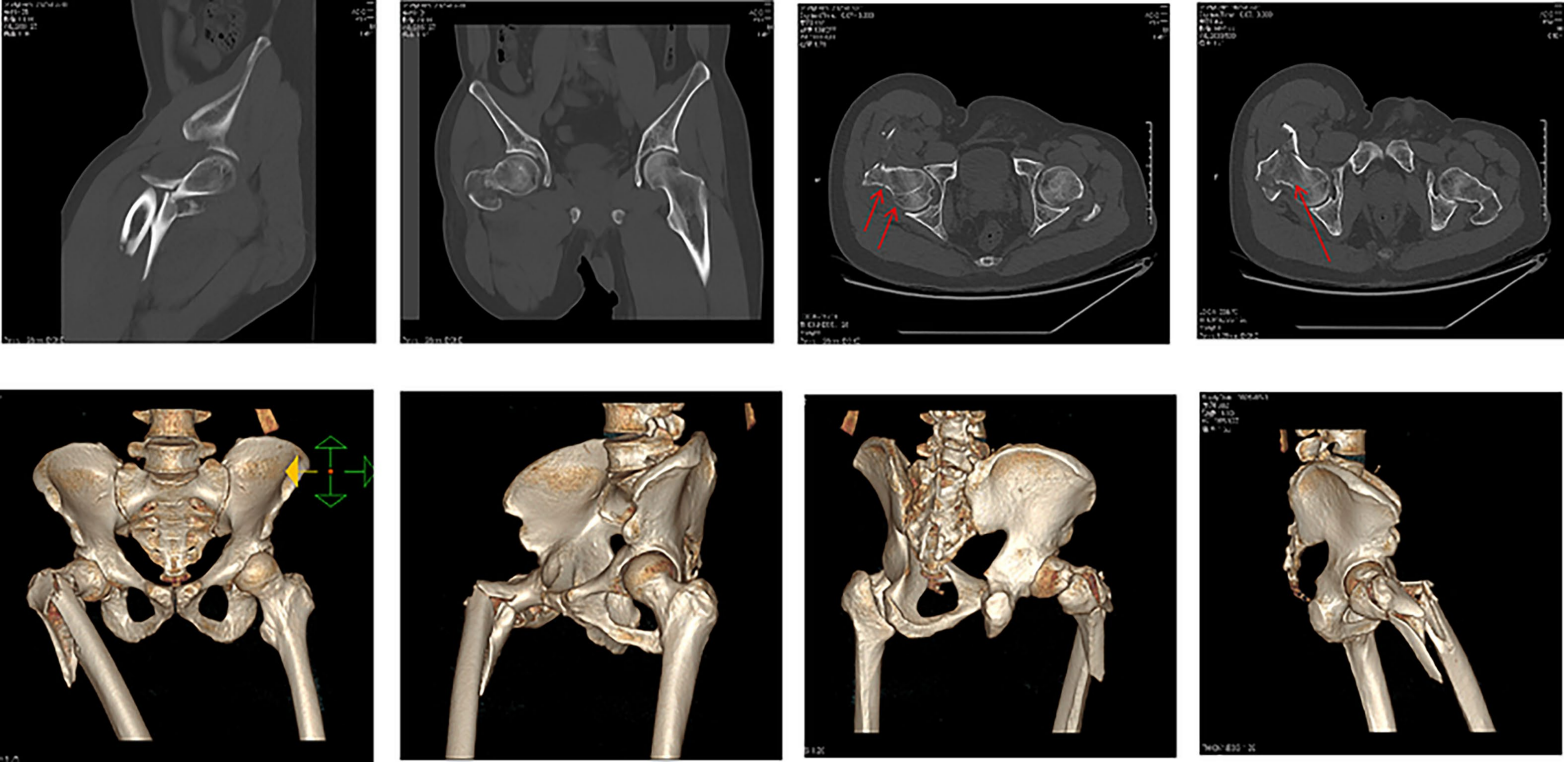
Preoperative pelvic CT scan demonstrating a subtrochanteric fracture with an inter-trochanteric component (Frank–Seinsheimer Type V).

**Figure 2:**
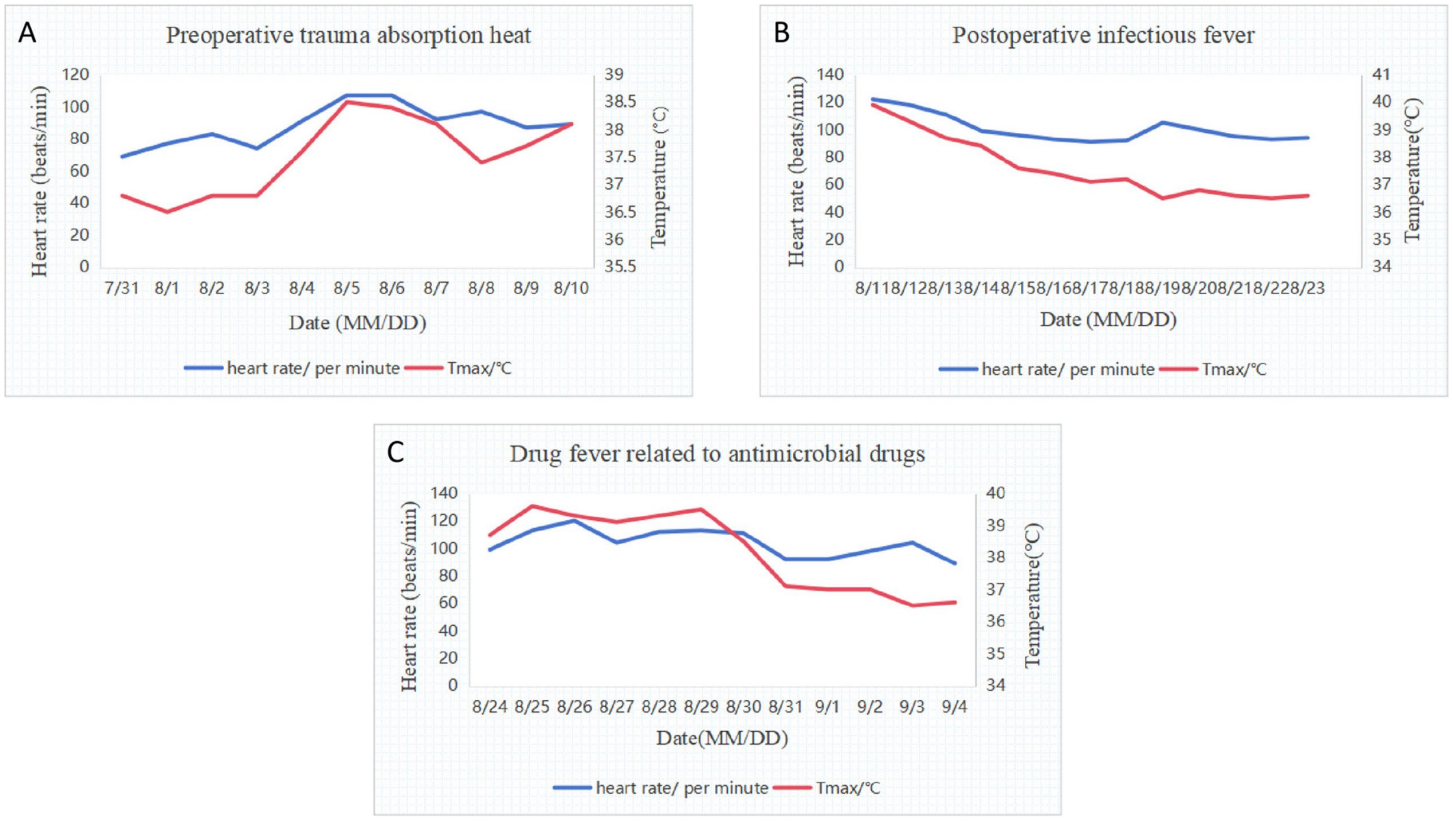
The body temperature curves demonstrate three types of fever during hospitalization: **(A)** Post-traumatic absorption fever; **(B)** Postoperative infectious fever; **(C)** Drug fever (antimicrobial-associated).

A 49-year-old male patient was admitted due to “pain and restricted movement in the right hip for 4 h.” His medical history included incomplete rib fractures and a lumbar vertebral compression fracture. Physical examination revealed swelling in the right hip with significant tenderness and axial percussion pain. Pelvic CT showed a comminuted fracture of the right femoral neck and intertrochanteric region (Frank-Seinsheimer Type V) (Figure 1). From admission until the scheduled surgery, the patient developed low-grade fever (36.8–38.5 °C) with mild elevation of inflammatory markers (WBC, CRP) (Table 1; Figure 2A). Blood cultures were negative. In light of the trauma history, the initial clinical impression was “post-traumatic absorption fever.” However, to prevent potential infection, empiric intravenous cefuroxime sodium was initiated on August 5.

**Table 1:** Laboratory tests during hospitalization.

Laboratory tests	8/1	8/5	8/9	8/10	8/11	8/12	8/13	8/14	8/15	8/17	8/20	8/24	8/26	8/28	8/30	9/1	9/7
WBC (3.5–9.5 10^9/L)	12.04	14.6	14.94	12.84	**23.31**	**17.41**	**16.83**	11.99	11.22	15.54	10.26	**11.14**	**10.17**	**4.31**	**3.1**	**6.5**	**9.67**
Neutrophils (40–75%)	70.6	77.4	70.1	72.4	**87.3**	**77.6**	**79.1**	68.7	70.1	74.1	63	**78.4**	**75.9**	**46.5**	**23.6**	**40.6**	59.5
Lymphocyte (20–50%)	22.1	15	18.1	18.6	7	13.2	10.2	18.5	19.9	19.4	25	11.5	14.3	41.2	56.8	38.3	26.4
Monocyte (3–10%)	6.7	6.7	9.6	7.3	5.4	8	8.6	8.7	7.1	5.7	9	8.7	8.2	11.9	18.7	15.4	11.1
Eosinophils (0.4–8%)	0.4	0.8	1.2	1.1	0.2	0.9	1.9	3.7	2.7	0.2	1.5	0.9	1.4	0.1	0.6	5.2	2.1
Basophils (0–1%)	0.2	0.1	1	0.6	0.1	0.3	0.2	0.4	0.2	0.6	1.5	0.5	0.2	0.3	0.3	0.5	0.9
RBC (4.3–5.8 10^12/L)	4.89	4.38	4.4	4.22	3.89	**2.4**	**2.48**	**2.64**	3.58	3.85	4.24	4.4	4.3	4.36	4.42	4.3	4.94
HGB (130–175 g/L)	108	98	97	96	89	55	55	85	85	90	101	103	100	103	101	97	109
PLT (125–350 10^9/L)	210	258	368	354	355	288	297	303	408	524	547	333	226	180	200	285	528
AST (15–40 U/L)	30.2	–	–	–	79.7	–	43.7	40	-	37.4	22.1	22.5	–	33.6	114	118.4	41.2
ALT (9–50 U/L)	29.5	–	–	–	75.5	–	47.7	49.4	-	83.2	53.1	30.4	–	20.7	42.7	83.8	86.1
GGT (10–60 U/L)	24.8	–	–	–	103.5	–	60.7	71.2	-	127.5	88.3	62.5	–	47.9	67	74.3	70.3
IL-6 (0–6.4 pg./mL)	–	24.51	–	–	–	**169.9**	**136.2**	18.31	13.04	3.02	5.65	42.99	14.03	8.13	8.03	5.71	–
hs-CRP (0-6 mg/L)	9.44	18.68	26.2	17.31	12.73	**123.41**	**123.68**	66.63	37.9	12.03	3.2	5.65	13.95	20.56	19.06	7.12	–
PCT (0–0.5 ng/ml)	–	0.107	–	–	0.276	**0.955**	**0.703**	0.371	0.258	0.102	0.073	0.179	0.301	0.672	0.614	0.27	–
ESR (0–16 mm/h)	13	36	58	53.00	–	**51**	**114**	87	83	58	34	23	11	23	21	23	–

WBC, white blood cell count; RBC, red blood cell count; HGB, hemoglobin; PLT, platelet count; AST, aspartate aminotransferase; ALT, alanine aminotransferase; GGT, gamma-glutamyl transferase; IL-6, interleukin-6; hs-CRP, high-sensitivity C-reactive protein; PCT, procalcitonin; ESR, erythrocyte sedimentation rate. Bold values denote laboratory results exceeding the upper limit of normal reference ranges.

### Operative period and early postoperative infection crisis (August 11, 2025 to August 23, 2025)

**Figure 3:**
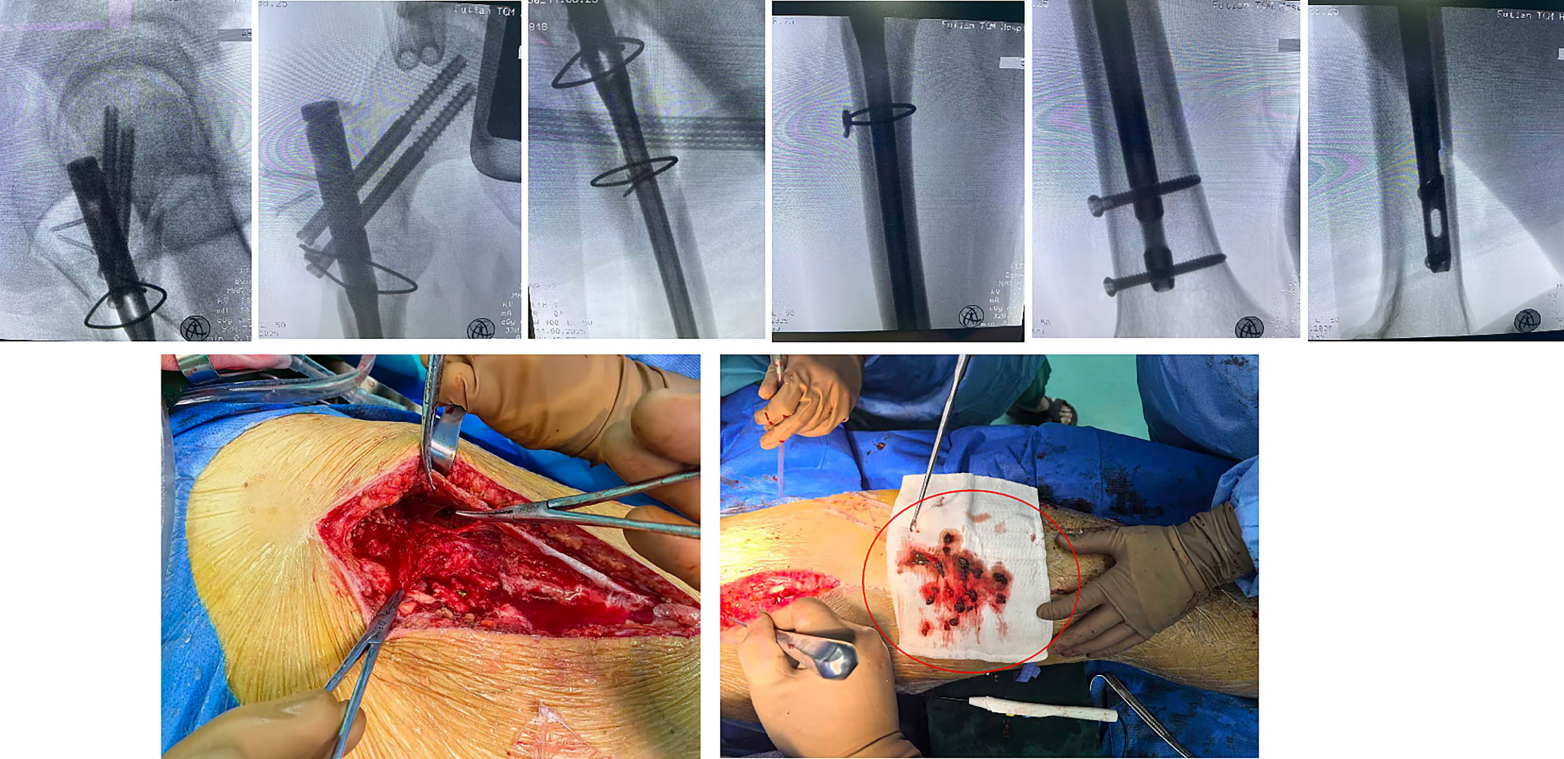
Intraoperative view. The upper part illustrates the position of internal fixation; the lower part demonstrates necrotic changes in an area of approximately 15 cm × 7 cm involving the tensor fasciae latae and the fascial layer of the vastus lateralis.

Given the unstable right femoral Seinsheimer type V fracture, surgery was performed on August 11, 2025, to reconstruct medial support, provide stable fixation, and reduce the risk of implant failure. The procedure was “open reduction and titanium cable fixation, intramedullary nailing, and debridement of pathological tissue for right femoral fracture.” Intraoperative exploration revealed a necrotic area (approximately 15 cm by 7 cm) involving the fascia of the tensor fasciae latae and vastus lateralis muscles. The necrotic tissue was debrided and sent for pathological examination (Figure 3).

**Figure 4:**
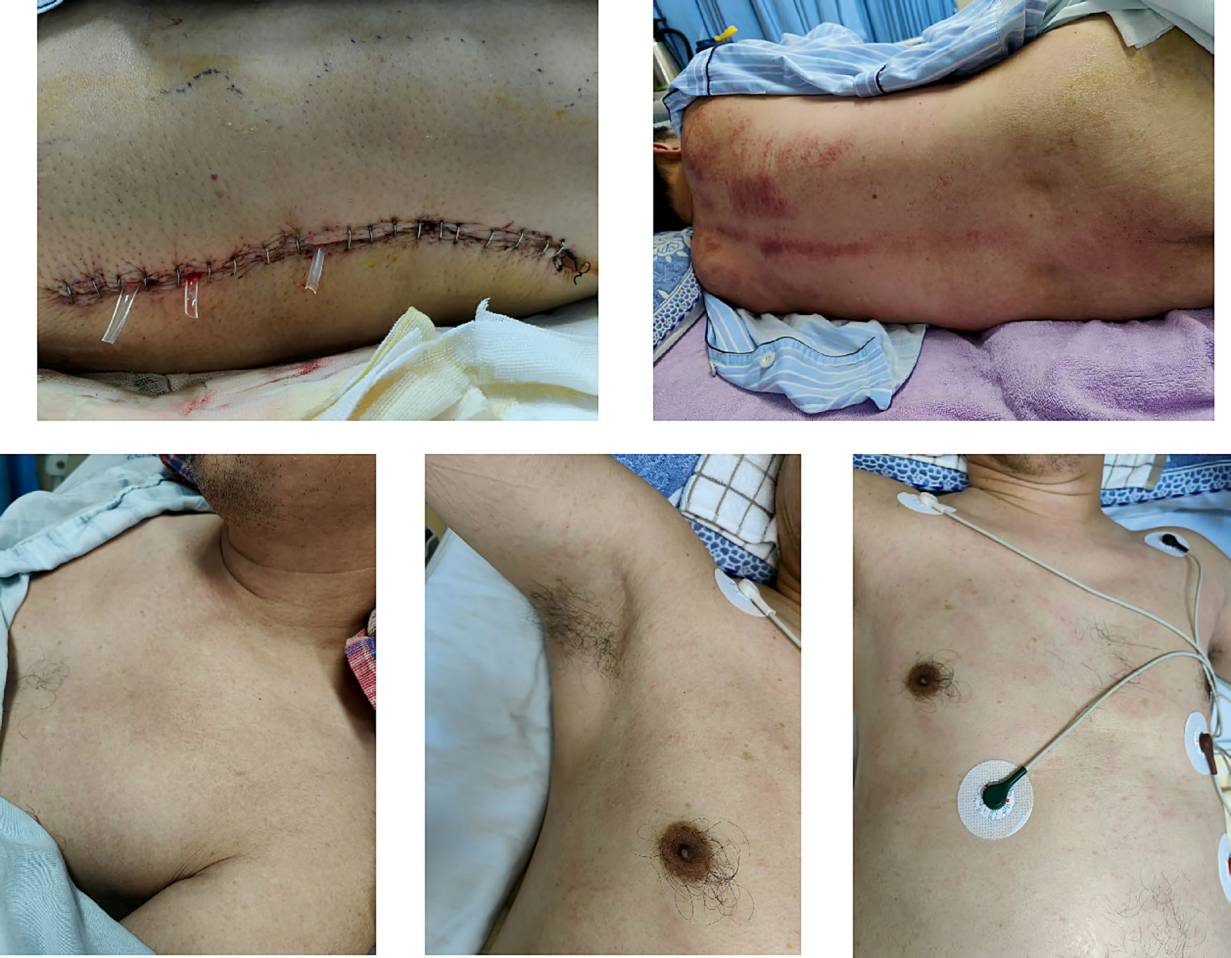
Skin and surgical site images.

Although postoperative fever was anticipated, the intraoperative finding of fascial necrosis, combined with the following acute changes, raised a high suspicion of necrotizing fasciitis and prompted an urgent diagnostic workup. On the evening of surgery, the patient’s body temperature spiked to 39.9 °C, accompanied by a sharp deterioration in inflammatory markers (WBC, CRP, ESR, IL-6, PCT) (see Table 1; Figure 2B). The pathology report indicated “acute and chronic inflammatory cell infiltration with tissue degeneration and necrosis.” Physical examination showed slightly darkened skin and mild pitting edema around the surgical wound, but no redness, exudate, or subcutaneous crepitus (Figure 4). Considering the severe systemic inflammatory response and the intraoperative findings, the possibility of necrotizing fasciitis could not be ruled out. Consequently, on August 13, the antibiotic regimen was escalated to a triple combination: piperacillin-tazobactam 4.5 g Q8h (August 12–30), clindamycin 0.6 g Q8h (August 13–26), and vancomycin 1 g Q12h (August 13–27), all administered intravenously. Vancomycin was infused at 10 mg/min, with serum trough concentrations of 3.96 mg/L (August 15) and 3.93 mg/L (August 26), both well below the target range of 10–20 mg/L. Clindamycin was added for anaerobic coverage and to suppress bacterial exotoxin production (e.g., streptococcal and staphylococcal toxins), a critical adjunct when necrotizing fasciitis is suspected. Blood transfusion was also administered to correct severe anemia.

After multidisciplinary discussion (involving the intensive care unit and infectious disease teams) and imaging evaluation (MRI showed minimal fluid collection without a definite abscess cavity or gas), and observing that the patient’s swelling subsided while inflammatory markers (PCT, IL-6, CRP) demonstrated a significant downward trajectory following the combined treatment, it was decided to postpone further surgical debridement and continue with conservative anti-infective management. The decision to pause surgical intervention was thus based on a combination of clinical improvement, trending laboratory data, and imaging findings that ruled out progressive necrotizing infection. On August 16, the patient developed scattered rashes in both axillae, upper limbs, chest, and abdomen, accompanied by mild pruritus. This was considered an allergic reaction to the antimicrobial agents, and intravenous dexamethasone (10 mg/d) was administered on August 16–17 for anti-allergy treatment, after which the rashes resolved. Subsequently, under this combination antibiotic regimen, the patient’s body temperature and inflammatory markers gradually declined, and his condition stabilized.

### Recurrent fever and the diagnosis and management of drug fever (August 24, 2025 to August 31, 2025)

After approximately 1 week of clinical stability, a new fever pattern emerged starting August 24: regular high-grade fever occurring daily from afternoon to night, with temperatures fluctuating between 37.3 °C and 39.6 °C, while remaining essentially normal during other periods (Figure 2C). This was accompanied by facial and cervicothoracic skin flushing, generalized scattered rashes with pruritus, and submandibular lymphadenopathy (Figure 4).

To identify the cause of fever, a systematic etiological and imaging workup was conducted. Repeated blood cultures yielded negative results. Although serial inflammatory markers showed mild elevation, comprehensive pathogen screening (including for fungi, viruses, *Mycobacterium tuberculosis*, and malaria parasites) revealed no definitive abnormalities. Further imaging evaluations (CT, MRI, and ultrasound of the chest, heart, urinary system, right hip, and abdomen) also failed to identify a clear infectious focus. Ultimately, ultrasound-guided aspiration of fluid collection in the right thigh was performed, and the sample was sent for metagenomic next-generation sequencing (mNGS), which similarly detected no definitive pathogen nucleic acid sequences.

In the absence of clear microbiological evidence, a review of the clinical course revealed a high temporal correlation between fever onset and drug administration (fever developed in the afternoon following morning intravenous antibiotic infusion). Furthermore, the presence of classic “vancomycin infusion syndrome”-like manifestations (rash, pruritus, facial and neck flushing) and hematological abnormalities (complete blood count on August 30 showed leukopenia at 3.10 × 10^9^/L and severe neutropenia at 0.73 × 10^9^/L) strongly suggested antimicrobial-induced “drug fever” with bone marrow suppression. A stepwise drug discontinuation strategy was therefore implemented: clindamycin was stopped on August 26, followed by vancomycin on August 27. As high fever persisted, piperacillin-tazobactam was finally discontinued on August 30. Notably, the patient’s body temperature normalized on the afternoon of the same day and remained stable without recurrence. The rash, skin flushing, and lymphadenopathy gradually subsided thereafter. The patient was successfully discharged on September 5.

## Discussion

Management of perioperative fever in fracture patients represents a core challenge that spans the entire diagnostic and therapeutic process, characterized by significant temporal evolution and etiological complexity. This case illustrates a complete etiological chain evolving from a preoperative non-infectious inflammatory response, to suspected severe deep infection in the early postoperative period, and ultimately to adverse drug reactions from antimicrobial agents. It provides a valuable reference for understanding the dynamic differential diagnosis of perioperative fever.

### Preoperative fever: revisiting post-traumatic systemic inflammatory response and “absorption fever”

The low-to-moderate grade fever (36.8–38.5 °C) observed in this patient preoperatively, accompanied by mildly elevated white blood cell count (WBC) and C-reactive protein (CRP) but negative blood cultures, and in the absence of signs pointing to respiratory, urinary, or other infections, should primarily be considered as post-traumatic “absorption fever.” In essence, “absorption fever” constitutes a systemic inflammatory response syndrome (SIRS) triggered by significant trauma, representing a nonspecific defensive reaction of the body to tissue injury (2). Tissue damage from trauma (e.g., decomposition of hematoma and necrotic tissue at fracture sites) releases damage-associated molecular patterns (DAMPs) (3). These molecules, including histones, high-mobility group box 1 (HMGB1), S100A8/A9, and mitochondrial DAMPs (mtDAMPs) (2, 4, 5), activate the immune system, drive the inflammatory cascade, and stimulate downstream signaling pathways such as NF-κB, MAPK, and cGAS-STING. This, in turn, promotes the synthesis and release of large quantities of pro-inflammatory cytokines by immune cells, including interleukin-1β (IL-1β), interleukin-6 (IL-6), and tumor necrosis factor-*α* (TNF-α) (6). These inflammatory mediators act on the hypothalamic thermoregulatory center, causing fever and an increase in acute-phase proteins. Temporally, such fever typically occurs within 24–72 h post-trauma, often presenting as remittent or irregular fever, with temperature fluctuations rarely exceeding 39 °C (7, 8). It is noteworthy that the trauma-induced inflammatory response is biphasic. While the initial SIRS aids in clearing necrotic tissue, it may be accompanied by a compensatory anti-inflammatory response syndrome (CARS), leading to secondary immunosuppression and increased susceptibility to infection (9). In this case, despite the empirical use of cefuroxime for infection prophylaxis, fever and inflammatory markers continued to fluctuate. This indicates that antimicrobial agents alone have limited efficacy in controlling non-infectious inflammatory responses. It also alerts clinicians that beneath the manifestation of “absorption fever,” continuous and dynamic monitoring is essential to remain vigilant for potential occult infections or the risk of the inflammatory response transitioning into infectious sepsis. The latter is typically accompanied by a more abrupt rise in inflammatory markers or the emergence of local signs.

### Postoperative early acute fever: differentiating infectious crisis from localized fascial necrosis

The differential diagnosis of fever in the early postoperative period following fracture surgery focuses on several key considerations, including surgical stress response, deep tissue infection, early implant-related infection, and thromboembolic events (e.g., pulmonary embolism) (10–13). In this case, the patient’s temperature spiked to 39.9 °C on the evening of surgery, accompanied by a dramatic worsening of inflammatory markers (WBC 23.31 × 10^9^/L, IL-6169.9 pg/mL, PCT 0.955 ng/mL). This signaled a shift in the nature of the fever from a non-infectious inflammatory response to a systemic infection/sepsis reaction. Temporally, the onset of sudden high fever within 72 h postoperatively necessitates primary consideration of acute surgical site infection. In terms of fever pattern, the patient exhibited a continued high fever, with temperatures persistently above 39 °C, consistent with a severe bacterial infection. Beyond the high fever, although the local wound showed no significant redness or exudate, the presence of darkened skin and pitting edema around the incision suggested that the infection was likely located at the deep fascial level rather than being a superficial incisional infection.

Procalcitonin (PCT) provided crucial discriminatory value here. Unlike broadly elevated inflammatory markers such as CRP and IL-6, PCT shows a more pronounced and specific increase in bacterial infections, particularly systemic ones (14). The patient’s PCT level was notably elevated at 0.955 ng/mL (>0.5 ng/mL), strongly indicating a bacterial infection rather than simply post-traumatic absorption fever. The intraoperative finding of ecchymosis and necrosis in the deep fascial layer, confirmed by postoperative pathology revealing “acute and chronic inflammatory cell infiltration with tissue degeneration and necrosis,” substantiated the presence of “fascial necrosis with infection.”

This condition must be distinguished from fulminant necrotizing fasciitis. The latter is a surgical emergency characterized by rapid, spreading insidiously infection along fascial planes, often accompanied by skin necrosis, marble-like skin discoloration, subcutaneous crepitus, and systemic toxic shock (15). The absence of these typical features in this patient, along with MRI findings showing no subcutaneous gas or extensive abscess formation, led to the diagnosis of “localized fascial necrosis with infection.” Although this localized infectious focus was sufficient to trigger a severe systemic septic response, it was effectively controlled through intraoperative debridement and a systemic antibiotic regimen (the triple combination of clindamycin, piperacillin-tazobactam, and vancomycin), thereby preventing severe consequences. This outcome underscores the importance of an anti-infective management strategy based on broad pathogen coverage and thorough intraoperative source control.

### Late-onset postoperative fever: the diagnostic challenge of drug fever and reflection on antibiotic stewardship

Drug fever is a common yet frequently overlooked cause of postoperative fever in orthopedics. Its diagnosis relies primarily on exclusion and typically requires meeting key criteria, including a temporal correlation between fever and drug administration, exclusion of other infectious or non-infectious etiologies, and prompt resolution of fever upon drug withdrawal (16). In this case, after initial control of infection markers and approximately 1 week of stable body temperature, the patient developed a new fever pattern in the second postoperative week (starting August 24). Temporally, this fever episode was distinct from the initial infectious phase, occurring during broad-spectrum antibiotic therapy. In terms of pattern, it exhibited a characteristic “drug-related” pattern with regular high fever occurring daily from afternoon to night, with peak temperatures reaching 39.6 °C, while pre-medication morning temperatures remained essentially normal. Regarding associated symptoms, the patient developed polymorphous rashes, facial and neck flushing, pruritus, and submandibular lymphadenopathy, with progressive leukopenia/neutropenia appearing later in the course. Comprehensive and repeated etiological investigations, including blood cultures and metagenomic sequencing, along with imaging evaluations, all yielded negative results. At this juncture, the differential diagnosis shifted decisively towards non-infectious causes, with the core challenge lying in distinguishing between infection recurrence and drug fever. Ultimately, the stepwise discontinuation strategy (clindamycin → vancomycin → piperacillin-tazobactam) led to normalization of body temperature within 24 h after the cessation of piperacillin-tazobactam, serving as the definitive “gold standard” for diagnosis. While this temporal relationship strongly implicates piperacillin-tazobactam as the primary culprit, it is important to acknowledge that the patient had received multiple antimicrobials concurrently; therefore, the reaction likely reflects a cumulative effect of combined drug exposure, with piperacillin-tazobactam being the predominant sensitizing agent and vancomycin potentially exerting a synergistic role.

Hypersensitivity reactions induced by piperacillin-tazobactam typically present as a delayed-type (Type IV) reaction, primarily mediated by T lymphocytes. The core mechanism involves the drug acting as a hapten that binds to host proteins to form a complete antigen. After processing by antigen-presenting cells, this antigen activates specific T lymphocytes, leading to the release of large quantities of pyrogenic cytokines such as interleukin-6 (IL-6) and interferon-gamma (IFN-γ). These cytokines act on the hypothalamic thermoregulatory center, causing systemic symptoms like fever, and are often clinically accompanied by signs of T-cell infiltration in the skin, such as eosinophilia and maculopapular rash (1). A severe manifestation of this T-cell-mediated delayed reaction is Drug-Induced Hypersensitivity Syndrome/Drug Reaction with Eosinophilia and Systemic Symptoms (DIHS/DRESS). This syndrome is a serious systemic adverse reaction characterized by fever, rash, internal organ involvement (e.g., hepatic or renal impairment), and hematological abnormalities (e.g., eosinophilia, atypical lymphocytosis) (16–18). Studies indicate that such reactions usually occur days to weeks after drug initiation, and their development may be related to immune interactions involving the reactivation of latent viruses (e.g., HHV-6) (18). For diagnosing this type of delayed hypersensitivity, traditional skin tests are of limited value. Research by Roehmel et al. (19) demonstrated that the Lymphocyte Transformation Test (LTT) holds significant diagnostic value for such reactions, exhibiting superior sensitivity and specificity compared to skin tests, further confirming the critical role of the T-cell-mediated mechanism in piperacillin-tazobactam-associated delayed hypersensitivity.

To further characterize the nature of this hypersensitivity reaction, we retrospectively applied the RegiSCAR criteria for Drug Reaction with Eosinophilia and Systemic Symptoms (DRESS). The patient’s presentation yielded a total score of 5 [fever ≥38.5 °C: +1; lymphadenopathy involving two or more areas: +1; rash >50% BSA: +1; liver enzyme elevation >2 × upper limit of normal: +1; exclusion of other causes: +1; eosinophilia >0.7 × 10^9^/L: 0 (peak eosinophil percentage was only 1.9%, corresponding to approximately 0.18 × 10^9^/L); atypical rash morphology: 0; renal involvement: 0; duration >15 days: 0]. A score of 4–5 corresponds to a “possible” case of DRESS. Thus, while the presentation falls within the spectrum of drug-induced hypersensitivity syndromes and shares several features with DRESS (fever, rash, lymphadenopathy, hepatic involvement), it does not fulfill the criteria for a definite diagnosis. This nuanced assessment reinforces the importance of maintaining a high index of suspicion for drug fever even when classic DRESS criteria are not fully met, particularly in patients receiving prolonged combination antibiotic therapy.

Beyond the classic T-cell-mediated reaction, piperacillin-tazobactam may also induce two others, often overlooked, adverse effects. The first is immune hemolytic anemia, which should be considered in the differential diagnosis of severe postoperative anemia (20). The second relates to the clarified controversy regarding the nephrotoxicity of piperacillin-tazobactam in combination with vancomycin. Research by Miano et al. (21) showed that while this combination is associated with a significantly increased risk (approximately 34%) of creatinine-defined acute kidney injury (AKI), it did not lead to an elevation in cystatin C—a more reliable marker of renal function. Furthermore, no significant changes were observed in patient dialysis rates or mortality (21). These findings suggest that the observed rise in creatinine likely reflects a “pseudo” interference of piperacillin-tazobactam with the creatinine assay, rather than indicating true tubular damage. Clinically, it is crucial to distinguish this effect from the genuine nephrotoxicity inherent to vancomycin.

The adverse reaction profile of vancomycin exhibits a “dual-pathway” characteristic. The most common is “vancomycin infusion syndrome,” a non-immunologic, pharmacologic reaction resulting from direct drug stimulation of mast cells and basophils, leading to massive histamine release. This reaction involves the activation of the Mas-Related G Protein-Coupled Receptor-X2 (MRGPRX2), triggering mast cell degranulation. The onset of vancomycin infusion syndrome is closely related to the infusion rate, manifesting as rapidly developing facial and neck flushing, pruritus, and urticaria. In severe cases, it can progress to angioedema, bronchospasm, and hypotension. While these symptoms mimic a Type I hypersensitivity reaction, they occur in the absence of IgE involvement. The reaction can often be mitigated or prevented by slowing the infusion rate (22, 23).

The other pathway is a true Type I, IgE-mediated hypersensitivity reaction, immunologically similar to that of piperacillin-tazobactam but mediated by T-cells. This mechanism involves vancomycin acting as an antigen triggering the production of IgE antibodies, leading to cross-linking of IgE receptors on mast cells and basophils and subsequent degranulation and histamine release. Unlike vancomycin infusion syndrome, this reaction is immunologically memory-based, independent of infusion rate, and can lead to more severe systemic reactions such as anaphylaxis (22). Additionally, vancomycin may directly suppress bone marrow hematopoiesis or cause neutropenia through an immune-mediated mechanism (23).

In summary, the “drug-associated” regular high fever, polymorphous rash, and hematological suppression observed in this patient during broad-spectrum antibiotic therapy represent a complex clinical presentation. This was likely driven by a T-cell-mediated delayed hypersensitivity reaction triggered by piperacillin-tazobactam (potentially compounded by vancomycin), in conjunction with histamine-release effects possibly associated with vancomycin.

In this case, the decision to maintain rather than escalate the antibiotic regimen after August 13 was supported by multidisciplinary consultation, favorable trending of inflammatory markers (PCT decreased from 0.955 ng/mL to 0.102 ng/mL by August 17), and MRI findings that excluded abscess or gas formation. These objective stop-points guided the conservative surgical approach and prevented unnecessary repeat debridement. Fracture-related infection (FRI) and surgical site infection are common postoperative complications. Contemporary antibiotic stewardship emphasizes timely de-escalation or discontinuation of therapy once infection is controlled, aiming to balance infection prevention against the risks of antimicrobial resistance and adverse drug events. In clear alignment with World Health Organization (WHO) recommendations, prolonging postoperative antibiotic prophylaxis (e.g., beyond 24 h) does not reduce the risk of surgical site infection but instead leads to unnecessary antibiotic exposure. Nevertheless, this practice of extended use remains widespread globally, particularly in low- and middle-income countries. Direct evidence supporting the WHO stance comes from a multicenter clinical study, which demonstrated that prophylactic antibiotic use beyond 24 h postoperatively did not lower surgical site infection rates but was associated with an average hospital stay prolonged by 1.4 days (24). Another randomized clinical trial focusing on antibiotic therapy following internal fixation for lower limb bone tumors revealed that a 5-day postoperative regimen (compared to shorter courses) significantly increased complication risks, such as drug-related adverse events (with a higher relative risk), underscoring the necessity of shortening prophylactic antibiotic use (25).

Furthermore, numerous studies indicate that antibiotic de-escalation therapy can reduce the risks of resistance and adverse drug reactions. Antimicrobial Stewardship Programs (ASPs) explicitly promote the principle of discontinuing antibiotics when they are no longer needed or switching from broad-spectrum to targeted agents (26). A large-scale retrospective cohort study highlighted that de-escalation of beta-lactam antibiotics is associated with a reduced incidence of new Gram-negative bacterial resistance, particularly in septic patients (27). Research by Kollef et al. (28) further supports the necessity of aggressive de-escalation based on microbiological results, especially given the rising threat of antibiotic resistance, to mitigate the potential harms of prolonged broad-spectrum therapy.

## Conclusion

This case illustrates a potential multi-stage etiological chain of fever in the perioperative period of fracture patients: beginning with non-infectious SIRS (absorption fever) triggered by trauma/surgical stress, progressing to sepsis induced by localized tissue necrosis with infection, and ultimately culminating in drug fever attributed to a cumulative hypersensitivity reaction to combination antimicrobial therapy, with piperacillin-tazobactam being the primary sensitizing agent. It highlights the following key clinical implications.

First, dynamic differential diagnosis is paramount. This necessitates integrating the fever’s temporal pattern, characteristics, and accompanying symptoms with a serial interpretation of inflammatory markers. Second, precise antibiotic stewardship is a double-edged sword. While initial empiric therapy requires broad coverage and high potency, decisive de-escalation or evaluation for discontinuation should be undertaken promptly upon obtaining clinical or microbiological evidence of control, strictly avoiding unnecessary prolonged broad-spectrum treatment. Third, a high index of suspicion for drug fever is essential. For any patient who develops a new, regular fever pattern during or after anti-infective therapy—especially when accompanied by rash, cytopenia, and a lack of clear infectious evidence—drug fever should be prioritized in the differential diagnosis and confirmed through a rigorous drug withdrawal trial.

In summary, the management of perioperative fever in orthopedics is far from a simplistic “fever-antibiotics” paradigm. It constitutes a complex decision-making process requiring collaborative, ongoing assessment and precise judgment by surgeons, infectious disease specialists, and intensive care teams. This case report provides a reference for this process, underscoring the critical role of diagnostic reasoning and antimicrobial stewardship in optimizing patient outcomes and minimizing complications.

## Data Availability

The raw data supporting the conclusions of this article will be made available by the authors, without undue reservation.
